# Two Coils Resonant Ramsey’s Method for the Measurement of Time Reversal Invariance Violation in Neutron Transmission

**DOI:** 10.6028/jres.110.072

**Published:** 2005-08-01

**Authors:** V. V. Loukachevitch, A. V. Aldushchenkov

**Affiliations:** St. Petersburg Nuclear Physics Institute RAD, 188350, Gatchina, Leningrad District, Russia

**Keywords:** asymmetries, density matrix, evolution operators, Ramsey’s method, scattering amplitude, T-violation

## Abstract

It is proposed within the framework of Ramsey’s method to register two-dimensional spectra, depending on the neutron phase and neutron energy, for measuring parity (P) and time (T) violating amplitudes of the interaction of polarized neutrons with polarized ^139^La nuclei in region of the *p*-wave resonance. The form of the phase spectrum and corresponding expressions for the asymmetries are obtained on the basis of a formalism of a spin density matrix. It is shown that the ratio of the P,T,-violating to P-violating imaginary amplitudes can be obtained from the measurements of the neutron phase spectrum with polarized and unpolarized ^139^La target.

## 1. Introduction

Whether or not the Standard Model (SM) describes charge-parity (CP) violation completely or there are additional sources of CP-violation beyond the framework of SM is an open question. Answers to this question may be found in studies of K, B meson decays and also in searches for CP- or T- violation in other areas of physics.

As the CP (or T) violating phase of the Cabibbo-Kobayashi-Maskawa (CKM) matrix is associated with the sector of heavy quarks, in the field of low energy physics the expected mechanism of T-violation is connected with meson exchange between nucleons [1]. The measured value of the T-violating amplitude in neutron transmission experiments is important in order to improve the value of the isovector pion coupling constant.

The most attractive experiment in this plan is the search for T-violation in a two-level system like^139^La where in the vicinity of the *p*-wave resonance, the P-odd effect is enhanced by 5 or 6 orders of magnitude [2]. Similar enhancement is expected for the interaction breaking P-, T-invariance [3]. The problem has been discussed in the literature for more than 20 years, but until now no realistic proposals of such an experiment have been formulated.

Traditionally, the scattering amplitude of polarized neutrons with a polarized nucleus is described as follows:

$$f=A+p_{t}B\left(\mathrm{sI}\right)+C\left(\mathrm{sk}\right)+p_{t}D\left(s\left[\mathrm{kI}\right]\right).$$ (1)

Here ***I*** is spin of a nucleus, *p*_t_ is polarization of a target, ***s*** is a neutron spin, and ***k*** is its wave vector. We note that the degree of target polarization can be rather high. So, for example, in Ref. [4] polarization of approximately 50 % is obtained with a (15 × 15 × 4) mm^3^ crystal.

There are a number of proposals on the determination and measurement of the weak amplitude *D* [5–11]. The measurement of this amplitude is an asymmetry in the counting rate of neutrons polarized with and against the direction of the T-violating field. However, according to the analysis in Ref. [12, 13], the physical realization of these experiments is extremely difficult. The known theoretical limit [1,2] *D* ≤ 10^−4^ *C*, obtained from modern restrictions on the neutron electric dipole moment [14], can be reached in proposed experiments. These experiments propose achieving an unprecedented (about 10^−4^) angular accuracy in the orientation of the neutron polarization vector and the requirement that the efficiencies of analyzer and polarizer are equal at the level of 10^−4^.

A completely different approach without the specified difficulties and consisting in refusal from the exact orientation of neutron spins concerning a direction of the T-violating field, gives the modified resonant method of oscillating fields. This method allows an increase in the number of degrees of freedom to operate the neutron spectrum.

## 2. Ramsey’s Method

The resonant two-coils Ramsey’s method [15] is widely applied to measurements of pseudomagnetic fields [16] and updating is reduced to the registration of a phase of a radio-frequency field at the moment of neutron detection.

Let us define a system of coordinates in which the external magnetic field and the initial polarization vector of the neutrons are directed along the axis *z*. Neutrons propagate along an axis *y* and an axis *x* is the direction of the vector product [***k*** × ***I***].

The amplitude of a radio-frequency field and the length of the coil can be chosen in such a manner that spins of the neutrons having the energy of a *p*-wave resonance *E*_0_ = 0.734 eV flip on $\frac{\pi }{2}$, and they are guided in *x-y* plane perpendicular to an external magnetic field. At random distribution of field phases δ, which we shall associate with the phase of a neutron, spins with equal probability in a direction are distributed in this plane.

The initial density matrix is diagonal and the components of the polarization vector in the chosen system of coordinates are equal:

$$p_{z}=p_{0},=p_{x}=p_{y}=0.$$

We define as well the geometry of the experimental setup. The length of each coil is equal to *l*; the length of a target is equal to *d*; and *L* is distance between each of coils and a target. Let us select the gyromagnetic ratio equal to unity. In this case the neutron spin precession frequency *ω* is equal to the value of magnetic field *H*. The Hamiltonian for a neutron in the magnetic field is given by:

$$H=\frac{\omega }{2}\left(n\sigma \right)=\frac{\omega }{2}\sigma_{z}.$$ (2)

Here ***n*** is the unit vector directed along the magnetic field.

For effective fields of a target, we shall accept definitions and designations as in Ref. [12]. The spin dependent part of amplitude in Eq. (1) characterizes the effective field vector ***b*** with the following components: a T-violating field $b_{x}=p_{t}\frac{D}{2}$, a field of weak interaction $b_{y}=\frac{C}{2}$, and a pseudomagnetic field $b_{z}=p_{t}\frac{B}{2}$. *ReB* represents the residual magnetic field, that is, the difference between the real part of a pseudomagnetic field of a target and an external magnetic field.

The target Hamiltonian can then be written as:

Hint=−iImA2−q(n1σ). (3)

In this expression the spin precession frequency in an effective field is $q=\sqrt{\mathrm{bb}}$ and the unit vector is $n_{1}=\frac{b}{q}$.

As is accepted in neutron optics, the effective fields are complex. Real parts of fields are responsible for a spin precession, and imaginary ones for absorption of neutrons in the substance of a target. The known Hamiltonian defines the corresponding evolution operator *U* = exp(−*iHt*), which will transform the density matrix. An expression for the evolution operator in a radio-frequency coil may be found in Ref. [17]:

$$U_{1,2}=\exp\left(-i\frac{\omega t_{1}}{2}\right)\exp(-i\frac{\omega_{1}t_{1}}{2}\left(n_{2}\sigma \right).$$

Here *t*_l_ is the neutron flight time through the coil, and the unit vector ***n***_2_ is determined by a phase δ of a radio-frequency field at the moment a neutron enters a coil. The components of this vector are given by:

$$n_{2x}=\cos\;\delta ,n_{2y}=\sin\;\delta ,n_{2z}=0.$$

## 3. A Phase Spectrum of Neutrons and Counting Rate Asymmetries

The density matrix of neutrons propagating through the described system is calculated from the following expression:

$$\rho \left(\delta \right)=U_{2}U_{L}U_{\mathrm{int}}U_{L}U_{1}\rho_{0}U_{1}^{+}U_{L}^{+}U_{\mathrm{int}}^{+}U_{L}^{+}U_{2}^{+}.$$ (4)

Here the indices of evolution operators define, accordingly, the number of the coil, distance *L* from the coil up to a target, and *int* — interaction with a target.

The two-dimensional neutron spectrum is defined as follows:

N=exp(−ImAt)Tr(ρaρ). (5)

To define the direction of spin before target, one needs to know the density matrix. It has the following expression:

$$\rho_{L+1}=U_{L}U_{1}\rho_{0}U_{1}^{+}U_{L}^{+}=\frac{1}{2}\left\{1+p_{0}\left[\sigma_{x}\;\sin\;\omega_{1}\;t_{l}\;\cos\left(\delta +\omega t_{l+L}-\frac{\pi }{2}\right)+\sigma_{y}\;\sin\;ù\;t_{1}\;\sin\left(\delta +\omega t_{l+L}-\frac{\pi }{2}\right)+\sigma_{z}\;\cos\;\omega_{1}\;t_{l}\right]\right\}$$ (6)

*t*_l+_*_L_* is the neutron flight time through the coil, and the interval *L* up to the target, which is convenient to represent as *t_l_*_+_*_L_*. *T* is the flight time through the entire system.

Omitting the exponent in Eq. (5), which is responsible for the total loss rate due to absorption in the target and does not merit further interpretation, we obtain an expression for a phase spectrum of neutrons. It follows from Eq. (6) that for the $\frac{\pi }{2}$ coil the interval

$$-\omega \frac{T-t}{2}\leq \delta \leq -\omega \frac{T-t}{2}+\pi$$ (7)

corresponds to neutrons with the spins distributed in a semi-plane with a positive direction of the *x*-axis. Phases *δ* shift this interval on *π* that determines neutrons with spin directions in semi-plane *x* ≺ 0

Shift intervals in Eq. (7) on $\frac{\pi }{2}$ and $\frac{3\pi }{2}$ will set the intervals with spin directions in semi-planes *y* ≻ 0 and *y* ≺ 0, accordingly.

Integration of a phase spectrum on the specified intervals gives values *N*_+_, *N*_−_, allowing one to calculate the asymmetry in the counting rate of neutrons connected with an axis *x* (a direction of vector product [ ***k*** × ***I***]) and an axis *y* (direction ***k***).

ΔNx=N+x−N−x=4sinω1tl{Im(by′bz′*)(p0+pacos(ε+ωt))+Im(bx′cosqt)(p0−pacos(ε+ωt))−pasin(ε+ωt)[Im(bx′bz′*)+Im(by′*cosqt)+p0cosω1tl(Re(by′bz′*)+Re(bx′cosqt))]+p0pacosω1tl[Re(bx′bz′*)(1−cos(ε+ωt))−Re(by′cosqt)(1+cos(ε+ωt))]}, (8)

ΔNy=N+y−N−y=4sinω1tl{Im(bx′bz′*)(p0+pacos(ε+ωt)+Im(by′cosqt)(p0−pacos(ε+ωt))−pasin(ε+ωt)[Im(by′bz′*)+Im(bx′*cosqt)+p0cosω1tl(Re(bx′bz′*)+Re(by′cosqt))]+p0pacosω1tl[Re(by′bz′*)(1−cos(ε+ωt))−Re(bx′cosqt)(1+cos(ε+ωt))]}. (9)

Integration of a phase spectrum in limits from 0 up to 2π gives total number of neutrons: *N* = *N*_+_*_x_*+*N*_−_*_x_* = *N*_+_*_y_*+*N*_−_*_y_*,

N=12{|bx′|2+|by′|2+|bz′|2+|cosqt|2+p0pasin2ω1tl[(|bz′|2−|cosqt|2)cos(ε+ωt)+2sin(ε+ωt)Re(bz′*cosqt)]+p0pacos2ω1tl(−|bx′|2−|by′|2+|bz′|2+|cosqt|2)+2cosω1tl[Im(bx′by′*)(p0−pa)+Im(bz′*cosqt)(p0+pa)]}. (10)

The normalization factors in Eqs. (8–10) are the values *n* = *N*_0_ exp (− Im *At*), where *N*_0_ is the initial neutron flux.

## 4. Determination of the T-Violating Amplitude

Let us discuss the experimental conditions for the determination of a T-violating amplitude. We shall suppose that the degree of compensation of a pseudo-magnenic field by external field provides the following inequality

|qt|<<1,atwhichsin(Reqt)≈Reqtandsinh(Imqt)≈Imqt. (11)

This condition is satisfied if the residual field equals about 1 % of a pseudomagnetic field. For neutrons with the energy at the maximum of *p*-wave resonance, it is possible to choose the following options of experimental parameters:

$$\varepsilon +\omega t_{0}=\{\begin{array}{lll} \pi & or & \pi +2k\pi \\ 2\pi & or & 2k\pi \end{array},\omega_{1}t_{0l}=\frac{\pi }{2},$$ (12)

where *t*_0_ means time flight for neutron with energy of maximum of *p*-wave resonance *E*_0_.

At such parameters, the three last lines in Eqs. (8) and (9) convert in a zero, and it becomes very easy to define the T-violating amplitude in this case. But at a displacement of the neutron energy from *E*_0_, the suppression factors differ from zero, and one needs to examine backgrounds more carefully.

For estimations one uses the fact that in the maximum of the *p*-wave resonance *ReC* is close to zero, and *ImD* ≅ 10^−4^ *ImC* and in accordance with Eq. (11) *ReBt* ≅ 0.1. Then, in Eq. (8), it is possible to neglect terms in which the values are less than 10^−5^ *ImCt* and in Eq. (9) terms smaller than *ImCt* by two or more orders of magnitude. As a result of such a reduction, Eqs. (8) and (9) in expanded form give the following expression:

ΔNx{π2π}=4[(p0∓pa)pt(ReBImC−ReCImB)t2+(p0±pa)ptImDt±p0sin(ωΔt)ImCt] (13)

ΔNy{π2π}=4[(p0∓pa)pt2(ReBImD−ReDImB)t02+(p0±pa)ImCt]. (14)

Notice that in Eq. (14) the top signs allow one to define an imaginary part of a P-odd amplitude. We can estimate the contribution of the last term in Eq. (13), considering that in the area of a resonance maximum value, *ImC* is changed insignificantly. Averaging this term in limits from *t*_0_ − Δ*t* up *t*_0_ + Δ*t*, we shall obtain

sin(ωΔt)ImCt¯=ImCt0ωt03(Δtt0)2=ImCt0ωt012(ΔEE0)2.

In this equality *ωt*_0_ is angle of spin turn in a pseudo-magnetic field of a target. For 1 cm of La this angle, according to value of the pseudo-magnetic moment from Ref. [16], is 17 radian. The suppression factor in this expression is equal to 10^−6^ for the energy window 2 × 10^−3^ eV and increases with an increase in the energy interval. It means that the background from Im*C* can be taken into account if the energy resolution is not worse than between 0.3 % and 0.5 %.

Passing in Eqs. (13) and (14) to average values, we shall obtain a final expression for the determination of the imaginary part of the T-violating field.

ptImD¯ImC¯=ΔNx(π)¯ΔNy(π)¯[1−p0−pap0+paΔNx(2π)¯ΔNx(π)¯−2p0(p0+pa)2sin(ωΔt)ΔNy(π)¯ΔNx(π)¯]. (15)

Because the coefficient in square brackets, which depends on the difference in efficiencies of analyzer and polarizer, must be known with precise accuracy, it takes additional measurement for its determination. In the case of a target that is unpolarized and isolated from an external magnetic field, it follows from Eq. (9):

$$\frac{p_{0}-p_{a}}{p_{0}+p_{a}}={\left(\frac{\Delta N_{y}\left(2\pi \right)}{\Delta N_{y}\left(\pi \right)}\right)}_{\text{unpolar}}.$$ (16)

Equations (15) and (16) represent the essential idea of the method. We suppose exact knowledge of the phase shift between generators of coils and neutron energy.

Thus, from this analysis it follows that the measurement of the T-violating amplitude of interaction of the polarized neutrons with the polarized target is reduced to carrying out two experiments with a polarized and non-polarized target.

## 5. Conclusion

Two-dimensional phase spectra of neutrons with energy in the region of the *p*-wave resonance contain sufficient information for the determination of a T-violating amplitude. The requirements presented here to measure the system in the described method can be realized in feasible experiment. In particular, the precision compensation of a pseudo-magnetic field by an external magnetic field is not obligatory. It is sufficient to have compensation on the level of 1 %.

One feature of the method is the opportunity of obtaining integrated asymmetries in the neutron counting rate as in the direction of the T-violating field and in the direction of the P-odd field of weak interaction from the same phase spectrum. These two asymmetries define P- and T-violating amplitudes in the maximum of the *p*-wave resonance. For the determining the amplitude in full region of the resonance, it is necessary to take into account background contributions arising from interaction of neutrons with the pseudo-magnetic field and P-odd field of the weak interaction. In this case the imaginary part of the T-violating field is separated from four neutron spectra, two of which are measured with a non-polarized target.

## References

[1] Towner IS, Hayes AC (1994). Phys Rev C.

[2] Alfimenkov VP (1983). Nucl Phys A.

[3] Bunakov VE, Gudkov VP (1984). (Paris) Colloq.

[4] Hautle P, Iinima M (2000). NIM A.

[5] Stodolsky L (1986). Phys Lett B.

[6] Kabir PK (1982). Phys Rev D.

[7] Masuda Y, Vylov Ts D (1992). Proceedings of WEIN ‘92.

[8] Skoy V (1996). Phys Pev D.

[9] Serebrov AP (1993). JETP Lett.

[10] Pentilla SI (2002). Proceedings of ASAP 2002.

[11] Masuda T (2002). Proceedings of ASAP 2002.

[12] Lamoreaux SK, Golub R (1994). Phys Rev D.

[13] Bunakov VE, Novikov IS (2000).

[14] Smith KF (1990). Phys Lett B.

[15] Ramsey NF (1949). Phys Rev.

[16] Glättli H (1979). J de Phys.

[17] Abragam A (1961). The Principles of Nuclear magnetism.

